# Surface Characterization and Anti-Biofilm Effectiveness of Hybrid Films of Polyurethane Functionalized with Saponite and Phloxine B

**DOI:** 10.3390/ma14247583

**Published:** 2021-12-10

**Authors:** Nitin Chandra Teja Dadi, Juraj Bujdák, Veronika Medvecká, Helena Pálková, Martin Barlog, Helena Bujdáková

**Affiliations:** 1 Department of Microbiology and Virology, Faculty of Natural Sciences, Comenius University in Bratislava, Ilkovičova 6, 842 15 Bratislava, Slovakia; d1@uniba.sk; 2 Department of Physical and Theoretical Chemistry, Faculty of Natural Sciences, Comenius University in Bratislava, Ilkovičova 6, 842 15 Bratislava, Slovakia; 3 Institute of Inorganic Chemistry, Slovak Academy of Sciences, Dúbravská Cesta 9, 845 36 Bratislava, Slovakia; helena.palkova@savba.sk (H.P.); martin.barlog@savba.sk (M.B.); 4 Department of Experimental Physics, Faculty of Mathematics, Physics and Informatics, Comenius University in Bratislava, 842 48 Bratislava, Slovakia; veronika.medvecka@fmph.uniba.sk

**Keywords:** organoclays, polymer nanocomposites, photodynamic inactivation, antimicrobial surface, photosensitizers

## Abstract

The main objective of this work was to synthesize composites of polyurethane (PU) with organoclays (OC) exhibiting antimicrobial properties. Layered silicate (saponite) was modified with octadecyltrimethylammonium cations (ODTMA) and functionalized with phloxine B (PhB) and used as a filler in the composites. A unique property of composite materials is the increased concentration of modifier particles on the surface of the composite membranes. Materials of different compositions were tested and investigated using physico-chemical methods, such as infrared spectroscopy, X-ray diffraction, contact angle measurements, absorption, and fluorescence spectroscopy in the visible region. The composition of an optimal material was as follows: *n*_ODTMA_/*m*_Sap_ = 0.8 mmol g^−1^ and *n*_PhB_/*m*_Sap_ = 0.1 mmol g^−1^. Only about 1.5% of present PhB was released in a cultivation medium for bacteria within 24 h, which proved good stability of the composite. Anti-biofilm properties of the composite membranes were proven in experiments with resistant *Staphylococcus aureus*. The composites without PhB reduced the biofilm growth 100-fold compared to the control sample (non-modified PU). The composite containing PhB in combination with the photodynamic inactivation (PDI) reduced cell growth by about 10,000-fold, thus proving the significant photosensitizing effect of the membranes. Cell damage was confirmed by scanning electron microscopy. A new method of the synthesis of composite materials presented in this work opens up new possibilities for targeted modification of polymers by focusing on their surfaces. Such composite materials retain the properties of the unmodified polymer inside the matrix and only the surface of the material is changed. Although these unique materials presented in this work are based on PU, the method of surface modification can also be applied to other polymers. Such modified polymers could be useful for various applications in which special surface properties are required, for example, for materials used in medical practice.

## 1. Introduction

Polymer nanocomposites with anti-biofilm properties are currently a very promising topic of basic and applied research. Several reviews on the biomedical applications of clay/polymer nanocomposites analyzed a broad spectrum of issues and current problems related to these materials [1,2,3,4]. Some risks associated with the use of the nanocomposites were also named and analyzed in detail [5,6].

Polyurethane (PU) represents a group of polymeric materials that can be prepared from a relatively broad spectrum of components. This variety allows the synthesis of materials of very different physical properties used for a wide range of different applications. Properties of various PU-derived materials predetermine them for many applications in medical biology. An example is the use of PU in the manufacture of implants, for which biodegradable polymers appear to be most promising. In the case of such implants, modification with antimicrobial agents may have an added value [7,8]. Other examples are fine fabrics based on PU composites that can serve as materials for antimicrobial membranes and filters and, in the medical industry, as wound dressings [9].

The surfaces of PU composites (PUC) often exhibit antimicrobial properties, which have been described in several papers [10,11]. In the case of composites with organoclays (OC), the antimicrobial effect of the surface was related to the amount and type of alkylammonium cations used in the silicate modification. As a rule, a significant effect occurred at OC contents of about 5%. The modified materials were more effective in the eradication of both Gram-positive and Gram-negative bacteria [12]. The bactericidal properties of OC-modified PU surfaces can be considered as evidence that the active components (silicate and especially cationic surfactants) interact directly with the surface of microbial cells [13]. The main bioactive components are alkylammonium cations, which are used to modify layered silicate particles and represent the main components of biocidal properties [14]. The positively charged groups of quaternary ammonium salts are believed to interact with the negatively charged groups located in the cell wall or cytoplasmic membrane by which they bind to the composite material [12]. Rarely, antimicrobial properties have been attributed to PU alone and only in specific cases, for example, due to the presence of active groups as a part of the polymer chains [15]. Normally, the PU material is without any significant biocidal properties. The antimicrobial effect of cationic surfactants used to modify the surface of PU without a silicate carrier has also been described, which only confirms the important role of surfactants for the antimicrobial properties of more complex systems and PUC [16]. The role of surfactants has, of course, also been confirmed for nanocomposites of other types of polymers [17]. 

The formation of biofilms on medical devices, such as cannulas and catheters, poses a serious threat, which often complicates medical treatment. This form of survival promotes homeostatic interaction and stability among microorganisms forming a community [18]. Moreover, biofilm is protected by extracellular polymer substances (EPS) that prevent or significantly reduce the penetration of biocides or antibiotics [19]. One way to prevent biofilm formation is to modify polymeric materials [20]. In addition to conventional nanocomposites, multicomponent systems with several antimicrobial substances are also possible. OCs are characterized by the ability to adsorb various bioactive molecules and thus form ternary systems with even better antimicrobial properties. In some cases, the effects of such modified surfaces have also been demonstrated against otherwise resistant biofilm-forming pathogens [10,11]. In a few cases, PUCs were prepared that could, in a controllable manner, release biologically active molecules into the environment, thus providing the potential for the elimination of the initial stages of biofilm formation. An example is a polymer composite capable of releasing tetracycline molecules from silicate particles dispersed in the polymer matrix [21]. In the form of microfibers, a relatively large surface area and the release of a sufficient amount of active substance were achieved and, because of this, the material was designed for wound healing application.

The objective of this work was the development of ternary nanocomposite systems based on a PU matrix with a functionalized surface using organically modified layered particles of saponite (Sap). The material was based on two antimicrobial components: a cationic surfactant (octadecyltrimethylammonium, ODTMA) used to modify the Sap particles and necessary to achieve compatibility with the polymer and the photosensitizer phloxine B (PhB). The properties of the precursor in the form of OC films with adsorbed PhB molecules have been tested in a recent study [22] and the effect and photoactivity of PhB as a part of other similar materials were confirmed in other works [23,24]. It is not expected that photooxidation of the surface by the photosensitizer should significantly alter the structure and antimicrobial properties of the composites. Nothing similar was confirmed in experiments aimed at monitoring changes caused by aging, thermo-, and photooxidation of the PU surface [25]. In addition, a methodology for the targeted increase of the concentration of active components exclusively on the surface of the material was developed in this work to minimize changes in the polymer matrix. Earlier studies have shown that the dispersion of nanoparticles in PU can significantly alter the structure and properties of the polymer matrix itself [26]. Therefore, besides the determination of anti-biofilm properties tested on resistant *Staphylococcus aureus* strain, sufficient attention was also paid to the characterization of physico-chemical properties of the PUC surface, especially those that were functionalized with a photosensitizer PhB.

The literature published on this topic so far has focused on composites of various types, in which organoclay particles have been distributed throughout the whole polymer matrix. Depending on a filler and the properties of a polymer, the particles can form structures with either an exfoliated, intercalated, or segregated phase. To the best of our knowledge, the strategy of surface-selective polymer modification presented in this work has not yet appeared in previously published literature. With this type of modification, the polymer matrix does not change, retaining its original properties. This work presents an example of how a successfully applied method of composite synthesis led to materials with anti-biofilm properties.

## 2. Materials and Methods

### 2.1. Materials

Synthetic Sap (trioctahedral layered silicate, commercial name Sumecton SA) was purchased from Kunimine Ind. (Tokyo, Japan) and used as received. ODTMA bromide (product number: 359246, CAS: 1120-02-1) and PhB (product number: P2759, CAS: 18472-87-2) were purchased from Sigma Aldrich in analytical grade quality. PhB stock solutions were stored at 4 °C in dark conditions. The precursors of PU were the commercial product VARNISH-PU 2 KW of Isomat S.A. (Thessaloniki, Greece). The precursors were based on two components: an aliphatic, water-based emulsion of polyurethane resin and a hardener. The hardener was based on the reactive linkers and catalysts as oligomers of hexamethylene di-isocyanate (CAS: 28182-81-2) (45–70%) and hexamethylene-di-isocyanate (CAS: 822-06-0) (<1%). N-butyl acetate (CAS: 123-86-4) (20–40%) was used to adjust the viscosity of the mixture. Polyethylene glycol tridecyl ether phosphate (CAS: 9046-01-9) (1–2.5%) was used as a stabilizer of the water-based emulsion. 

### 2.2. Bacterial Strains

The standard strain *S. aureus* CCM3953 (Czech Collection of Microorganisms, Brno, Czech Republic) and a methicillin-resistant *S. aureus* (MRSA) L18 were tested in this study. The clinical isolate was acquired from central venous catheters (Institute of Microbiology, Faculty of Medicine, Comenius University in Bratislava, and the University Hospital in Bratislava, kindly provided by Prof. Lívia Slobodníková, Ph.D.). Both strains were cultured on Mueller–Hinton agar (MHA, Biolife, Milan, Italy) at 37 °C (thermostatic cabinet, Lovibonds, Biosan, Riga, Latvia) for 24 h. A single colony was picked and used for further overnight cultivation, which was used for anti-biofilm assays.

### 2.3. Synthesis of Modified and Functionalized Organoclay Films

**Figure 1:**
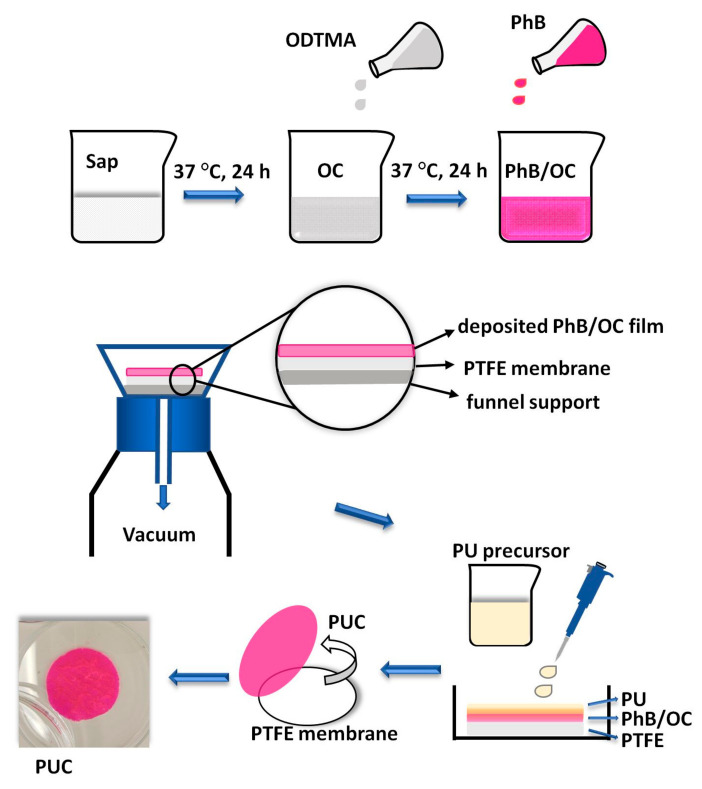
The schematic diagram on the preparation of polyurethane membranes with surface modified with phloxine B-functionalized organoclay. Sap—saponite, ODTMA—octadecyltrimethylammonium, OC—organoclay, PhB/OC phloxine B—functionalized organoclay, PTFE—polytetrafluoroethylene, PU—polyurethane, PUC—polyurethane composite (PU modified with PhB/OC).

The Sap films with adsorbed ODTMA cations and PhB molecules were prepared from colloidal dispersions (Figure 1). In the first step, colloidal dispersion of Sap was prepared by immersing Sap powder in deionized water (2 g L^−1^) and stirring for 30 min. ODTMA solutions of different concentrations were prepared in deionized water and heated on a hot plate stirrer until transparent solutions were formed. Sap colloidal dispersion was mixed with the heated ODTMA solution with shaking at 40 °C at 150 rpm for 24 h (Orbital Shaker-Incubator ES-20, Biosan, MI, USA). In the next step, an appropriate amount of PhB solution was added, and the mixture was shaken again under the same conditions. In preliminary experiments, different compositions of the colloidal dispersions varying the amounts of the components were used to search for optimal properties of the films (Table 1).

After 24 h, the colloids were filtered through membrane filters (0.1 μm Omnipore, hydrophilic PTFE, 47 mm diameter, Millipore, Merck). Initially, 20 mL of the colloid was rapidly filtered in the first step and then 5 mL was added in the second step to evenly spread the particles on the surface of the membrane. The theoretical concentration of deposited Sap was 0.8 mg cm^−2^. The supernatants were collected and measured by a UV/Vis spectrophotometer to control the amount of eluted PhB. The films of the deposited solids on the membranes were used for the preparation of the modified PU membranes (Figure 1). They are named in the following text as organoclays (OC) or PhB/OC to underline the presence of PhB molecules.

### 2.4. Preparation of PU Membranes with Functionalized Surfaces

The PTFE filter membrane covered with OC or PhB/OC films was moved to a Petri dish plate and layered with a liquid mixture of the PU precursors (0.013 mL cm^−2^). Following the procedure of the manufacturer, the resin and the hardener were mixed in a 4:1 *w*/*w* ratio under stirring to get a homogeneous and uniform emulsion. The liquid was able to form a transparent, thin PU membrane upon curing for about 120 min. The PU membrane was left to cure and then dry completely at room temperature (RT, 22 °C) under dark conditions overnight. The PU phase penetrated the layer of an OC or PhB/OC film. The following day, the PUC films with the surface modified with OC or PhB/OC were peeled off from the PTFE membrane (Figure 1) and used directly for physico-chemical characterization and anti-biofilm assay. The control samples with PU alone or PUC modified with OC (without PhB) were also prepared and tested. 

### 2.5. Preliminary Tests

Preliminary experiments have been focused on testing several membrane compositions. Important criteria were the amount of PhB incorporated into the membrane during synthesis and the stability of PUC in the medium used for the biological experiments. In the first step, membranes were already tested during PhB/OC film synthesis. By visual observation, it was possible to determine what proportion of the added dye was incorporated into the prepared PhB/OC films. The concentrations of added PhB solution and free PhB remaining in the supernatant after the preparation were compared. The percentage of PhB that was incorporated into the film relative to the total amount of dye added was determined. In the next step, PUC membranes of various compositions were prepared and tested using fluorescence spectroscopy. 

### 2.6. Interaction of Polyurethane Composite with the Medium Used in Biological Experiments

To characterize the release of the active substances from these membranes, their stability upon the interaction with a medium used for biofilm growth was further characterized to provide the final selection of the optimal membrane composition. The same conditions were applied for the biofilm growth. The series of M4 samples (three prepared membranes with three cut replicas each) were chosen for the characterization. 1 cm × 1 cm square pieces were cut from circular-shape membranes to represent middle and near-edge parts of the membrane circles. Fluorescence spectroscopy was directly applied for the characterization of PhB photoactivity in the membranes. Two groups of the samples were measured and statistically compared. Principal component analysis and multivariate curve resolution were applied to evaluate different properties of the compared series (see Supplementary data, Section S1). The release of PhB from the M4 membrane to supernatant was investigated in detail in another experiment. The membranes with known PhB content were immersed in MHB at 37 °C. After certain times, small amounts of supernatants were collected and, after dilution to a defined volume with deionized water, analyzed by absorption spectroscopy. By determining the PhB concentration using the Lambert–Beer law (*ε* = 8.4 × 10^4^ mol^−1^ cm^−1^ dm^3^), the percentage of PhB released from the membranes was calculated.

### 2.7. Physico-Chemical Characterization of Polyurethane Membranes

To evaluate the wettability of the surface, the contact angle measurements were carried out using a SEE System device (Advex Instruments, Brno, Czech Republic) with supported software for surface energy evaluation SeeSoft 7.0. The distilled water and diiodomethane were used as polar and non-polar liquids in the tests. The volume of droplets was 2 µL. The Owens–Wendt model was applied for the determination of surface energy with polar and dispersive components [27]. The attributed values were calculated from at least 10 measurements. The infrared spectra were obtained on a Nicolet^TM^ is50 Fourier transform infrared (FTIR) spectrometer equipped with IR source, KBr beamsplitter, and DTGS detector measurement using the built-in single-reflection ATR accessory with the diamond crystal (Thermo Scientific, Waltham, MA, USA). Collection of the spectra and other operations were performed using the Thermo Scientific OMNIC™ software package. X-ray diffraction (XRD) patterns were measured with the X’Pert PRO (PANalytical B.V., Almelo, The Netherlands) diffractometer equipped with CuKα radiation (*λ*_α1_ = 1.5406 Å) operating at 45 kV and 40 mA and PIXcel^3D^ solid-state hybrid detector. The measurement was in the range from 2 to 30° (2*θ*) with scanning step 0.02° and scanning step time 96.4 s. Diffuse reflectance spectra (DRS) of all the samples in the visible region were recorded at 300–800 nm by Varian UV-Vis spectrophotometer CARY 5000 (Agilent, Santa Clara, CA, USA) using Spectralon as a reference, equipped with PRAYING MANTIS^TM^ diffuse reflection system accessory (Harrick Scientific Products Inc., Pleasantville, NY, USA). All DRS spectra were transformed to absorption spectra by the Kubelka–Munk function. Steady-state fluorescence spectra of polymer films were recorded by Spectrometer Fluorolog 3 (Horiba Jobin Yvon, Palaiseau, France). The fluorescence intensity was detected in the front-face configuration. The spectra of all the films were recorded after excitation at 510 nm with the excitation and emission slits 0.75 nm and 1 nm, respectively. The excitation spectra were recorded at 640 nm with the excitation and emission slits 1 nm and 1 nm, respectively. All the XRD and spectra measurements were performed at room temperature.

### 2.8. Anti-Biofilm Assay by Photodynamic Inactivation

For the determination of anti-biofilm activity, three types of PU membranes were tested: non-modified PU (control), PUC membranes with the modified surface using OC, and those modified with PhB/OC hybrid particles. The “PhB/PUC” denotes the PU composite containing particles of OC functionalized with PhB. The composition of the membranes was based on the M4 type (Table 1). The membranes were cut into 1 cm × 1 cm squares and sterilized under UV light (Esco class 2 BSC, Singapore) for 15 min on both sides. For anti-biofilm assays, the squared pieces of PUC were stuck to the bottom of wells in a 24-well microtiter plate (Sarstedt, Nümbrecht, Germany) using 150 μL of MHA. The plate with the stuck membranes was UV-sterilized again, as stated above.

Bacterial cultures of *S. aureus* were grown in 40 mL of Mueller–Hinton broth (Biolife, Milan, Italy, MHB) overnight at 37 °C at 150 rpm in an incubator shaker. On the next day, the cultures were adjusted into OD_600_ 0.05 in MHB supplemented with 4% glucose (CentralChem, Bratislava, Slovakia) and continued culturing to OD_600_ 0.5 (i.e., 2 × 10^7^ cells mL^−1^). The 24-well microtiter plates with fixed membranes were covered with 500 µL of MHB containing cultures of *S. aureus* CCM 3953 or L 18 and supplemented with 500 µL MHB supplemented with 4% glucose. Plates were incubated in the dark at 37 °C for 24 h to form biofilms. Then, membranes were washed with 2 mL of 1× PBS (PBS; 137 mM NaCl, 2.7 mM KCl, 8 mM Na_2_HPO_4_, and 2 mM KH_2_PO_4_; CentralChem, Bratislava, Slovakia), and shaken for 2 min in an incubator shaker at 150 rpm (Orbital Shaker-Incubator ES-20, Biosan, Latvia). The non-modified PU membranes were used as the control sample. One set of biofilm formed on PhB/OC was irradiated with a green laser (*λ* = 532 nm, 100 mW, Alligator, MZTech s.r.o., Košice, Slovakia) for 120 s. After the irradiation, all membranes (also those not irradiated) were immersed in 10 mL 1× PBS and sonicated for 5 min at 55 kHz (Branson 200 ultrasonic cleaner, Danbury, CT, USA) then vortexed for 5 min and serially diluted and plated to MHA. The colony-forming units (CFU) were calculated after 24 h. Each sample was tested in triplicate, and the experiment was repeated twice. Results are expressed as average values with standard deviations. More details about the method have been published elsewhere [22].

### 2.9. Scanning Electron Microscopy of Biofilms

The samples with formed biofilms used for scanning electron microscopy (SEM) were prepared as described above. The membranes with biofilms were transferred to new 6-well polystyrene plates (Sarstedt, Nümbrecht, Germany) and washed with 2 mL of 1× PBS for 2 min and fixed in 4% paraformaldehyde (CAS No: 30525-89-4, Sigma-Aldrich, St. Louis, MO, USA) for 1 h in the dark. The samples were then washed twice with 1× PBS and sterile distilled water for 10 min each at room temperature (RT) before they were post-fixed in 1% osmium tetroxide (CAS No: 20816-12-0, OsO_4_) for 1 h in the dark. These samples were, again, washed twice in 1× PBS and sterile distilled water for 10 min each, followed by dehydration steps: washing in 25%, 50%, 75%, and 95% (*v*/*v*) ethanol/water solutions for 10 min at each step and twice in 100% ethanol for 15 min. The dehydrated samples were allowed to dry at RT. The fixed samples were then sputter-coated with carbon (20 nm) using a QISOT ES Sputter Coater (Quorum Technologies, Lewes, UK) and held with carbon tape on an SEM sample holder. SEM was viewed under a JSM-7100F electron microscope (JEOL, Tokyo, Japan).

### 2.10. Mathematical, Statistical, and Chemometric Methods

Simple mathematical tasks, such as curve fitting and statistics, were performed using OriginPro 17 software (OriginLab Corporation, Northampton, MA, USA). Chemometric methods principal component analysis (PCA) and multivariate curve resolution (MCR) were done using Unsrambler^®^X version 10.3 (Camo Software AS, Oslo, Norway). 

## 3. Results and Discussion

PhB is a member of halogenated xanthene dyes with photoactive properties and a high antimicrobial potential. It has been proved to be effective against Gram-positive bacteria, including MRSA strains [22,28,29,30]. Sap is a layered silicate with a negative layer charge and does not significantly adsorb PhB molecules without an appropriate modification [22,23,24]. Therefore, Sap particles had to be modified by ODTMA cations to activate the surface for PhB adsorption and in the same way to get a compatible material for the nanocomposite with PU. Samples with variable loadings of ODTMA and PhB were prepared, characterized in terms of physico-chemical properties, and tested against biofilm formed by Gram-positive bacteria of *S. aureus*. Our goal was to optimize the composition of the material to get a sufficient amount of adsorbed phloxine B. The prepared material should be stable and exhibit a high antimicrobial activity to prevent the formation of a biofilm.

### 3.1. Selection of Optimal Polyurethane Composite Membranes

Four types of hybrid films of Sap modified with ODTMA and functionalized with PhB were prepared and tested in preliminary experiments. In addition to a sample with an amount of ODTMA equivalent to cation exchange capacity (M4), OCs with an excess of surfactant were also tested (M-M3). The reason was to increase the number of positively charged ODTMA cations and to verify whether this factor can increase the effective adsorption of PhB anions. Not all samples were suitable as precursors for PU-functionalized membranes. Among the PU membranes assigned as M1–M4 (Table 1), M1 and M4 were ideal from the view of PhB adsorption as there was no significant elution/loss of the dye during the preparation of the PhB/OC films. The supernatant separated by the filtration from M1 and M4 films did not contain significant amounts of the dye. On the other hand, the red color of the supernatants indicated relatively low adsorption of the dye in M2 and M3 films (Table 2). ODTMA in moderate amounts played a positive role in the activation of Sap surface, leading to efficient adsorption of PhB anions. On the other hand, the presence of a high amount of ODTMA above the cation exchange capacity probably led to saturation of the silicate surface, which prevented efficient PhB adsorption. The formation of ternary systems can be understood as a process of competitive adsorption between ODTMA and PhB molecules. Therefore, the smallest amount of adsorbed PhB was observed for the M3 sample, starting from the system of the smallest PhB and the highest ODTMA concentrations (Table 1) not providing free sites for the PhB adsorption. Membranes M1 and M4, containing less ODTMA and having more free sites to absorb PhB, retained >98% of added dye (Table 2). Almost all PhB was retained in the M1 film. 

Organoclay films M1-M4 modified with PhB were used for the synthesis of PhB/PUC membranes. Fluorescence spectroscopy was used to evaluate PhB photoactivity in the membranes. In general, the emission intensity relates to the number of photoactive molecules and their efficiency at emitting light. The highest activity was observed for M4 films and decreased in order M4 (100%) > M2 (98.9%) > M3 (35.6%) > M1 (23.5%). The high value obtained for M2 is due to the twice larger loading of the dye used for the synthesis of this membrane (Table 1). Although not all dye was adsorbed in this sample (around 73%, Table 2), it was sufficient to achieve a relatively high luminescence. Lower fluorescence of M3 could be assigned to the lower amount of the dye on the membrane (Table 2). However, the lowest fluorescence of M1 could only be due to fluorescence quenching. A high amount of absorbed dye probably caused the formation of specific species, such as molecular aggregates, able to quench fluorescence [31]. M1 and M3 exhibited the shift of the emission peak to a higher wavelength (598 nm), indicating photophysical interactions between PhB molecules. It might have occurred due to the formation of molecular aggregates, which may relate to the composition of the membranes, such as the presence of ODTMA over the CEC of Sap, the structure of the intercalated phase, etc. These aspects were not analyzed in detail but helped to select the membrane type of optimal properties. M4 exhibited optimal properties when considering dye adsorption and PhB photoactivity. 

Significantly different properties between PhB/PUC membranes of M1 and M4 types were observed when performing pilot biological experiments. The membrane M1 with a higher amount of surfactant exhibited a significant and rapid release of the dye when interacting with the medium used for anti-biofilm growth (Section S2). As already mentioned, this type of membrane was also oversaturated with ODTMA with respect to the CEC (Table 1). It absorbed PhB most efficiently (Table 2), but the PhB/PUC complex was not very stable. The rapid and immediate release of the large fractions of the dye can be disadvantageous due to a rapid loss of active photosensitizer molecules from the surface. PhB was released in a much lower amount from the M4 membrane upon contact with the medium. Due to the low photoactivity, poor stability, and fast release of PhB molecules from the M1 membrane, the selection for further experiments was narrowed to the M4 type.

### 3.2. Phloxine B Release from Polymer Composite Membranes

**Figure 2:**
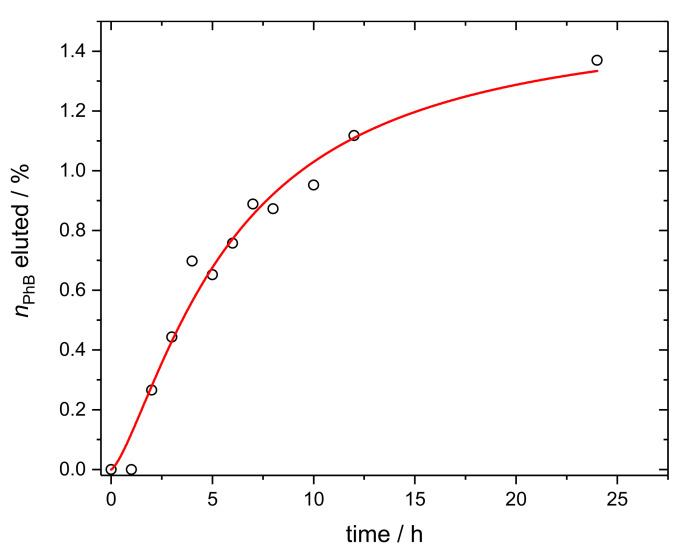
The elution of PhB from the membrane by the medium MHB. The elution was fitted by the logistic function $n_{\mathrm{PhB}\%}=n_{\mathrm{PhB}\%,\max}(1-1/{\left(t/t_{c}\right)}^{p})$ finding maximum of the released PhB $\left(n_{\mathrm{PhB}\%,\max}=1.52\pm 0.16\right)$ and time at the half-elution time *t*_c_ = 5.9 ± 1.1 h.

Partial, slow, and controlled release of the dye from the surface into the medium may also have its advantages in terms of antimicrobial activity. The released molecules can kill the cells of bacteria or even prevent them from settling on the membrane to form biofilm or at least reduce cell activity during this process. Therefore, the experiments focused on the release of PhB were carried out. In the first step, two series of samples (1 cm × 1 cm square pieces of membranes) were tested. The fluorescence of the PhB in the samples that were treated with the medium was compared with the untreated membranes. Chemometric analysis of the fluorescence spectral data of the samples of both series confirmed a smaller amount, i.e., lower fluorescence of PhB in the samples after the treatment with the medium (Section S3, Figure S2a,b). An experiment determining the release of the dye over time indicated a relatively stable binding of PhB in M4 membranes (Figure 2). Less than 2% of the dye was released into the medium over time, which was a negligible amount compared to the total dye content in the membranes. The course of the release was modeled using a logistic function from which it was possible to estimate the half-life of the process to approximately 6 h. This can only be considered as a rough estimate, as the exact characteristics of the dye release kinetics would require further measurements and more detailed analysis. The release of ODTMA tested using IR spectroscopy was not observed, confirming stable bonding of alkylammonium cations to Sap particles. 

### 3.3. Physico-Chemical Characterization of the Membranes

#### 3.3.1. X-ray Diffraction

**Figure 3:**
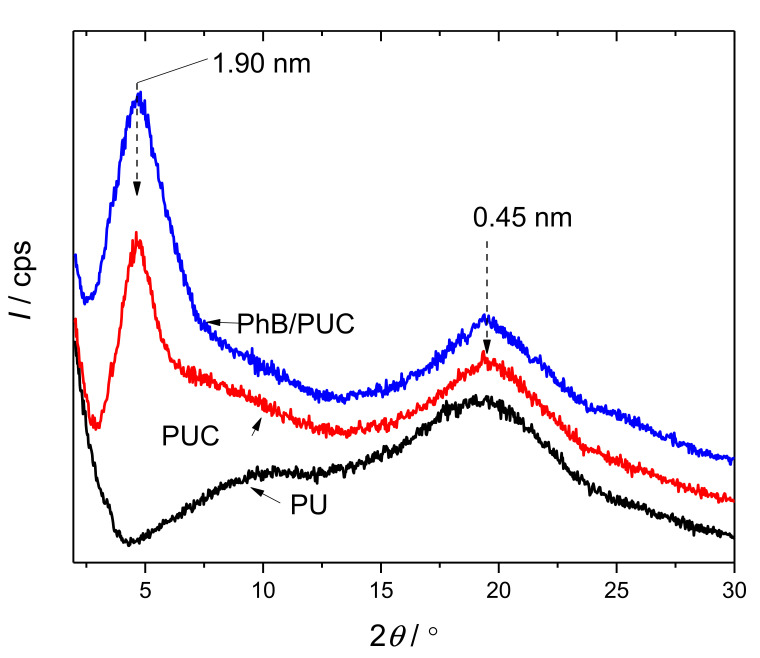
X-ray diffraction patterns of the membrane samples. Non-modified polymer (PU), PU composite modified using organoclay (PUC), and PUC modified with organoclay functionalized with phloxine B (PhB/PUC). Broad reflections at about 10 and 19.5° belong to the PU phase. The peaks near 5° are assigned to the 001 reflections of the organoclay phase.

A broad hump was observed in the profile of the XRD pattern of pure PU membrane near 19.5° (2*θ*), which relates to the spacing of about 0.45 nm (Figure 3). The shape of the reflection indicates an amorphous phase of the polymer and a low grade of crystallinity [32]. A broad reflection of 2*θ* ≈ 10–11° was assigned to a *d*-spacing of 0.85 nm associated with the reflections of the polymer phase, namely of the planes lying in regions perpendicular to the lamellar surface [33]. The signals of both polymer and an OC phase in the PUC specimen were detected for the modified surfaces (PUC and PhB/PUC). This means that both phases were present on the surface of the modified membranes, at least in terms of detectability by the XRD method. The modification with OC did not seem to affect the broad signals from the polymer matrix (2*θ* ≈ 10–11°, 19.5°), indicating the presence of OC did not significantly affect the structure of the polymer. A new, sharper reflection appeared in the patterns of the modified surfaces related to 1.9 nm *d*-spacing, assigned to the basal reflections from OC particles. This value fits well to the thickness of silicate layers plus the interlayer spaces occupied with ODTMA cations and possibly also intercalated polymer chains. It seems the presence of small amounts of PhB cations in complex PhB/PUC membranes did not affect the spacing significantly. This trend is similar to the results observed for PhB-functionalized organoclays published recently [22]. OC films used for the preparation of the PUC membranes were also measured (Section S4, Figure S3). Slightly but significantly lower values were observed (1.78 nm), indicating partial intercalation of polymer chains in the PUC samples, causing the expansion from 1.78 to 1.9 nm. 

#### 3.3.2. Infrared Spectroscopy

**Figure 4:**
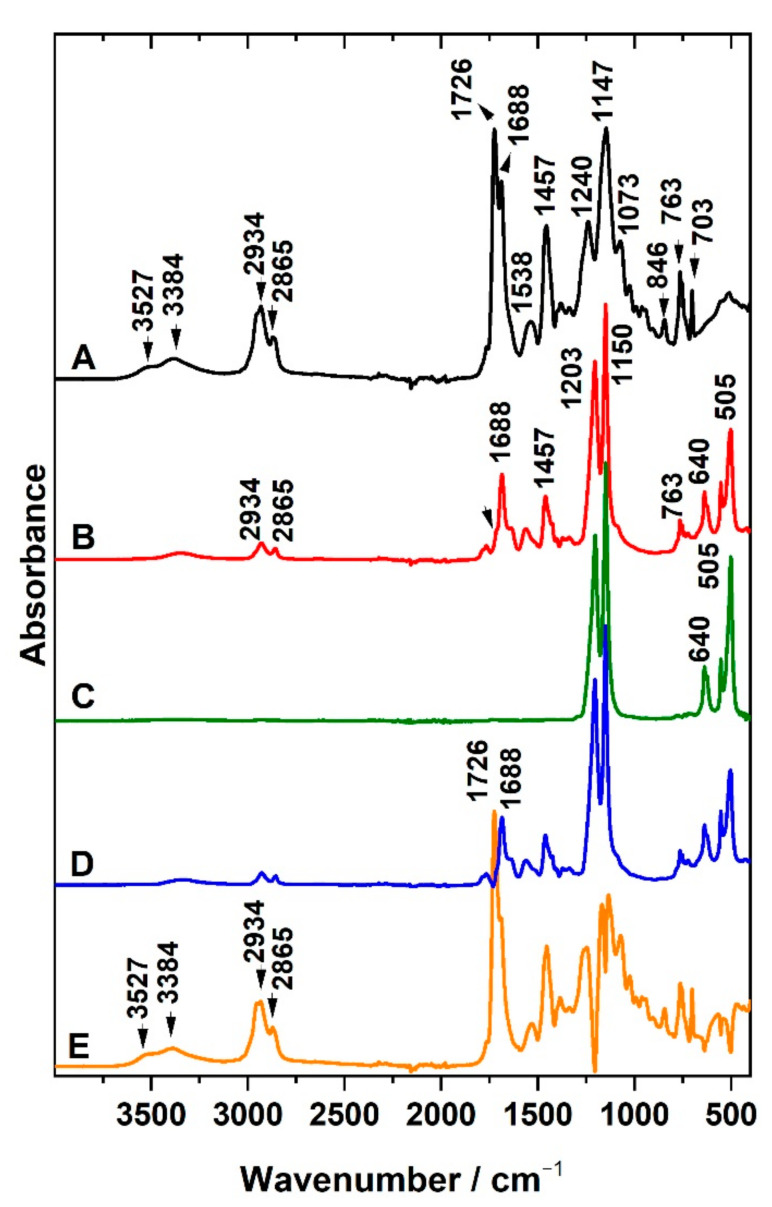
ATR spectra of non-modified polyurethane (PU) membrane (**A**), PU membrane from the contact side with PTFE (**B**), pure PTFE filter (**C**), and the spectral profile of components calculated by the MCR method (**D**,**E**).

Attenuated total reflection was used for the characterization of the surfaces of the prepared PU, PUC, and PhB/PUC membranes. As mentioned in the experimental part, liquid PU precursors were mixed and poured onto a PTFE filter membrane and, after the curing of the polymer, a PU membrane was obtained by peeling from the PTFE support. IR spectra of the non-modified PU membrane were investigated in the first step. The side of the PU membrane that came into contact with air during the curing only gave the spectrum typical for aliphatic PU (Figure 4, spectrum A). At the region 3000–2800 cm^−1^, a complex band with several components at 2955 (shoulder), 2934, 2865 cm^−1^ belongs to asymmetric and symmetric stretching C–H vibrations of methylene groups occurring in polyurethane chains. A broad band around 3384 cm^−1^ could be attributed to the N–H stretching vibrations. Since the membrane was cured starting from an aqueous emulsion, the band at 3527 cm^−1^ could be assigned to the OH stretching vibration of residual water molecules H-bonded to the polar groups of the polymer. The vibration of N=C=O groups at 2270 cm^−1^ expected to be dominant for the precursors was not detected, which means the reactants had all been consumed during the curing of the polymer. In the region below 2000 cm^−1^, the intensive bands at 1688 and 1726 cm^−1^ belong to characteristic bands of stretching vibrations of C=O functional groups, H-bonded as well as without H-bonds, respectively. They involved vibrations of C=O present in the functional groups of variously developed polymeric chains and fragments, including those of isocyanatane trimers. Bands of C–H bending vibrations from methylene groups occurred at 1457 cm^−1^ and N–H bending also called amide II band (trans-form) at 1538 cm^−1^. Typical bands of PU included C–C stretching and rocking vibrations of aliphatic groups in polymer chains at 1240, 1024, 846, and 763 cm^−1^, C–O stretching vibrations at 1073, 1147 cm^−1^, but also other types of C–H bending bands. Details on the band assignment of the PU membrane are also given in Table S1.

The measurement from the side which was in contact with the PTFE filter (Figure 4, spectrum B) gave the spectral profile different from that described for PU membrane (spectrum A). In addition to the bands assigned to PU, new strong bands appeared in the spectrum. It should be noted that the contribution of PU bands to this spectrum was lower and the intensity of the new bands dominated the spectrum. The same bands were detected in the PTFE filter membrane measured for comparison purposes (spectrum C). The spectrum of the PTFE filter is represented by bands assigned to CF_2_ bending vibrations at 505 and 554 cm^−1^, C–C–F bending vibrations at 640 cm^−1^, and CF_2_ stretching vibrations at 1203, 1150 cm^−1^ (Table S1), thus clearly confirming the presence of PTFE on the contact side of the PU membrane sample. The bands occurring between 1800–1300 cm^−1^ and the presence of vibrations of CH*_n_* groups in the range 3000–2800 cm^−1^ observed for the PTFE/PU side can be explained by the presence of PU forming the bulk of the membrane. A significant change occurred for relative intensities of both components of the characteristic C=O band (1688 and 1726 cm^−1^). The presence of a hydrophobic PTFE membrane may have played a role in the interaction with the different polar groups of the reactant molecules during the curing of the polymer. Chemometry was applied to confirm individual components in the complex spectra of prepared composites. For this purpose, a series of multiple measurements from several spots on both sides of three membranes prepared at identical conditions were carried out. The spectral matrices were analyzed using the MCR method. Two main spectral components were calculated. The first component (spectrum D) was almost identical to the spectrum measured for the side of the PU membrane in contact with PTFE (spectrum B). The second component (spectrum E) strongly resembled the PU spectrum (spectrum A).

The IR spectra analysis of both sides of the PU membrane, therefore, confirmed different chemical compositions. On the side of the previous contact between PU film with the PTFE filter membrane, the PU molecules penetrated the porous structure of the PTFE membrane before the curing of the polymer was completed. PTFE is a polymer that does not interact strongly with most substances, including PU; however, the porous structure of the PTFE membrane filter had caused mutual bonding between both components. Thus, the simplest membrane type, such as a PU membrane, exhibited different spectra on its different sides. This finding led to the decision that only the side that represented the pure PU could be used in the biological experiments as the control sample. 

**Figure 5:**
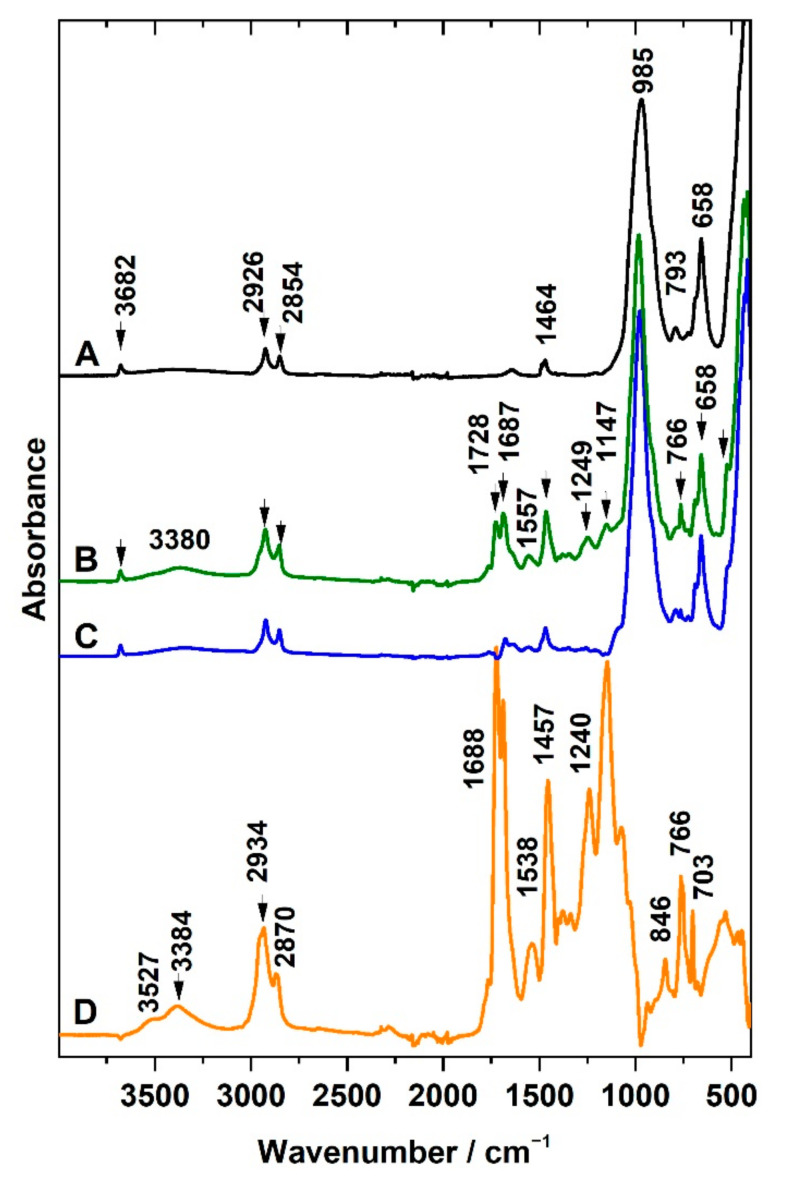
ATR spectra of organoclay OC (**A**), PUC membrane modified with an organoclay (**B**), and spectra of components calculated from the series of PUC-membranes spectra by MCR method (**C**,**D**).

IR spectra of surfaces modified with organoclay are introduced in Figure 5. Spectrum A represents the OC used for modification, while spectrum B represents the surface of the PUC membrane on the side where the modification of the polymer with OC film was done. In addition to bands previously attributed to PU (Figure 4, Table S1), the IR spectrum provides bands of OC composed from ODTMA and Sap. Synthetic Sap (layered silicate) can be identified based on the bands of stretching and bending vibrations of the structural OH group at 3682 cm^−1^ and 658 cm^−1^, respectively, and also based on Si–O bands. Stretching asymmetric Si–O vibrations are present as the complex band at 985 cm^−1^, while corresponding bending vibrations are exhibited at 460 cm^−1^. Other Si–O bands are present at 693 cm^−1^ [34]. The presence of ODTMA used for the modification of Sap is reflected in the presence of the bands attributed to stretching C–H vibrations from methyl and methylene groups in the spectral region 3000–2800 cm^−1^. Bending C–H vibrations can be found at 1457 cm^−1^. The most intensive bands of PU belong to stretching C=O vibrations at 1687 and 1728 cm^−1^. In general, the intensity of the bands of OC dominated over bands of PU in the spectra of PUC samples (Figure 5B). 

The MCR method was applied, analyzing the series of spectra obtained for several membranes to verify the identity of the spectral components (Figure 5). The calculated profiles C and D represented typical examples of the spectra of OC or PU, respectively. The latter was almost identical to that identified for non-modified PU (see Figure 4, spectrum A). An interesting observation is that no PTFE bands were detected in the spectrum of the modified membrane (Figure 5B). The curing of PU on the top of OC film led to the formation of a composite thin layer connected to the PU solid membrane. The presence of the OC film prevented the penetration of the polymer to the pores of the PTFE support. Some bands in the calculated spectrum D (Figure 5), representing a pure PU, were shifted with respect to the positions observed in spectrum B. For example, the shift of the band from 1464 to 1457 cm^−1^ can be assigned to various forms of aliphatic CH_2_ groups in both the surfactant and PU. A similar interpretation can be used for the shifts of CH_2_ stretching vibrations between wavenumber region 2870 and 2854 cm^−1^ and between 2935 and 2926 cm^−1^.

NIR spectroscopy was also applied to characterize the films. This method did not yield any significant results as it is less sensitive than spectroscopy in the middle IR region and was mainly able to detect bands corresponding to the PU matrix (Figure S5). It was not possible to distinguish the bands of the other components, nor by the MCR method, as the other components formed only a small proportion in the given materials and weak signals. PTFE is completely transparent in the NIR region, and all higher vibrations are detectable only in the middle IR region. Even the presence of organoclay on the surface of PUC membranes could not be recognized by NIR spectra from the PU bulk. On the other hand, it was possible to confirm the structure of the PU matrix showing the bands in the NIR region typical for this polymer, such as the overtones C–H, N–H, and C=O and their combination vibrations. Details on the assignments and a representative spectrum of the PU membrane are shown in S6. Small amounts of PhB in the functionalized PhB/PUC membranes did not allow to detection of the bands belonging to this substance in IR spectra in either of the IR regions.

#### 3.3.3. Phloxine Spectral Properties in the Visible Range

**Figure 6:**
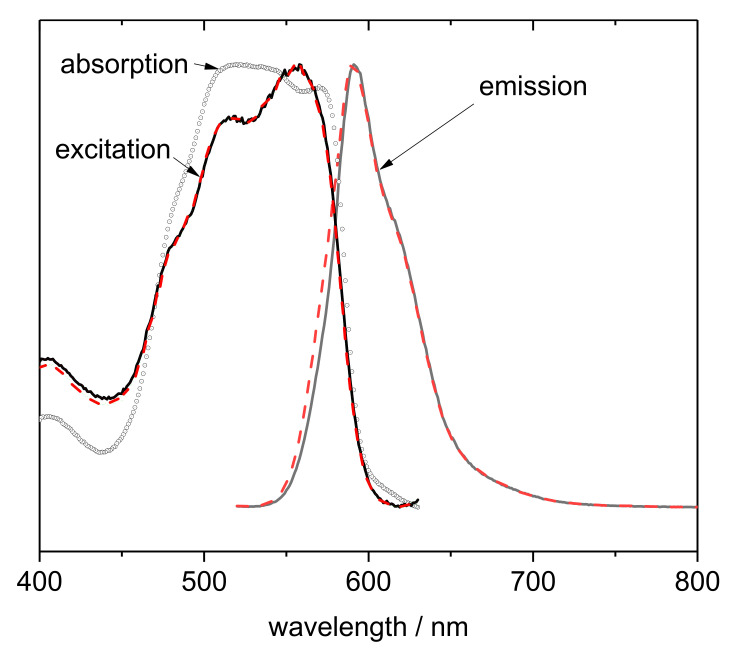
Spectra of polyurethane composite membrane modified with organoclay particles functionalized with PhB. The spectra were normalized to the maximum value.

Visible diffuse reflectance and fluorescence spectra were measured to characterize the spectral properties of PhB in the prepared PhB/PUC membranes. The luminescent properties have already been confirmed when comparing different types of membranes, as discussed in Section 3.1. Only the membrane of the M4 type is described in more detail here since it is the only one exhibiting a relatively high content of adsorbed PhB, good stability, and also relatively high photoactivity (see discussion in Section 3.1). The absorption, excitation, and emission spectra of the PhB/PUC membrane are shown in Figure 6. Only qualitative information can be expected from the absorption spectrum as it was calculated from the data measured in the diffuse-reflectance mode. The absorption band in the range of ~515–550 nm was relatively broad with an indistinct shoulder at 570 nm. For comparison, the band at 565 nm with the shoulder at 525 nm was measured in the previous work for the film of OC with adsorbed PhB [22], but the measurement had been performed in the transmission mode and, therefore, the spectrum was more accurate. In a previous study, lower wavelengths of band positions were observed for a dye solution (540 nm) and Sap colloids modified with polycations (546 nm), and a shift to higher wavelengths was observed for solid films prepared from these colloids (555 nm) [23]. The excitation spectrum of the PhB/PUC was relatively similar to the absorption spectrum (Figure 6). It was characterized by a band at 556 nm and two indistinct shoulders at 516 nm and 480 nm. A very similar spectrum with the bands at 558 and 523 nm was reported recently for the film of organoclay with PhB [22]. The emission spectrum of the PhB/PUC membrane gave the maximum emission at a relatively high wavelength (591 nm) (Figure 6). OC with PhB, as well as saponite-modified polyelectrolyte films published in previous studies [22,23], gave lower wavelength values of the emission maximum (582 and 567 nm). It is probable that, in the solid phase, photophysical interaction occurred due to the increased concentration of dye, which led to the red spectral shifts. Similar effects have already been observed in hybrids of PhB and poly (2-hydroxyethyl methacrylate) [35]. 

#### 3.3.4. Surface Properties

**Figure 7:**
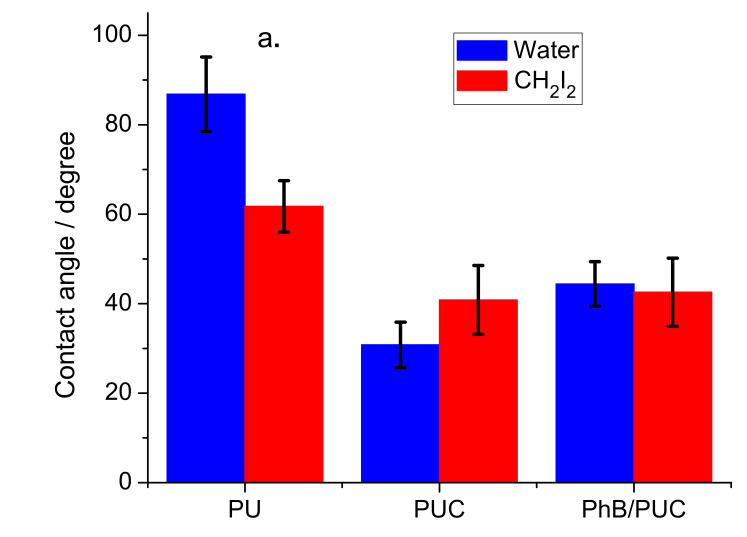

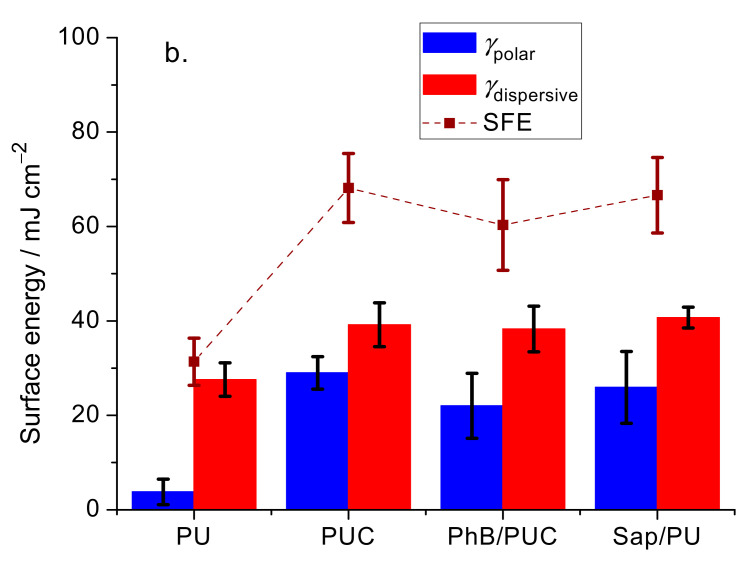
Water contact angles (**a**) and surface energy of PU membranes (**b**). PU—pure polyurethane, PUC—polyurethane composite with ODTMA/Sap particles (without PhB), PhB/PUC—PU composite with PhB.

Higher contact angles were determined for pure PU membrane regardless of the type of solvent: water (87° ± 8°) and CH_2_I_2_ (62° ± 6°) (Figure 7a). The PUC samples exhibited lower values (*p*-value < 0.001). The high contact angle of water on the PU membrane would indicate the hydrophobic properties of this polymer which is in agreement with some other studies [36,37]. A relatively high CH_2_I_2_ contact angle indicates that the PU membrane is not a typical hydrophobic material. The surface free energy (SFE) of this material is relatively low (31 ± 5 mJ m^−2^) (Figure 7b). Such properties can be partially explained by the surface topography of the polymer (Figure S6). The formation of the cavities can be explained by the procedure and starting materials used for the synthesis of the PU membranes. Curing started from the aqueous-based liquid precursors (emulsions) and residual water phase in the form of microdrops likely accumulated near the surface. The microcavities might have been formed after water and other volatile compounds had evaporated. The cavities were not observed on the surface of PUC membranes (not shown). The relatively large SFE values (60–68 mJ m^−2^) of PUC samples were due to polar (22–29 mJ m^−2^) and dispersive surface energies (38–40 mJ m^−2^). It is in contrast with the properties of the OC based on ODTMA and Sap with an SFE slightly below 50 mJ m^−2^ described in the previous study [22]. The surface properties of the PUC membranes described in this work can be explained by the process of synthesis of these membranes. A thin layer of OC coming into contact with the liquid precursor efficiently adsorbed water together with other volatile compounds, which prevented the formation of microcavities on the surface of a composite. Similar changes were observed for PU composites with present OC in another study reporting the reduction of contact angles for water, as well as non-polar solvents [37]. The trends of increasing energies with dispersed OC were observed also for other polymers. For example, a slight decrease in water contact angles was observed for polypropylene and polymethylmethacrylate with an increasing amount of OC [38,39]. Polystyrene composite with OC based on a fluorine-containing surfactant exhibited the reduction of water contact angle of the pristine polymer (91°) to the value of 32° for the composite [40]. In another work, the modification of poly(vinylidene fluoride) with OC led to the reduction of water contact angle to ~15° [41]. The surface topography, roughness, and the concentration of polar groups on the surface, including the silicate particles, are the most likely explanation for this phenomenon. Although the properties of PU and its composites are significantly different, the composite materials (PUC, PhB/PUC) exhibited relatively similar properties (Figure 7). Since non-modified PU exhibited the lowest surface energies, this could result in a weaker adhesion of the bacteria cells to its surface. On the other hand, PUC or PhB/PUC surfaces with higher surface energies can be relatively more accessible for cell anchoring and biofilm growth. In any case, the differences in surface energy between the compared samples should not lead to significant differences in biofilm growth.

### 3.4. Biological Experiments

#### 3.4.1. Anti-Biofilm Assays by Photodynamic Inactivation

**Figure 8:**
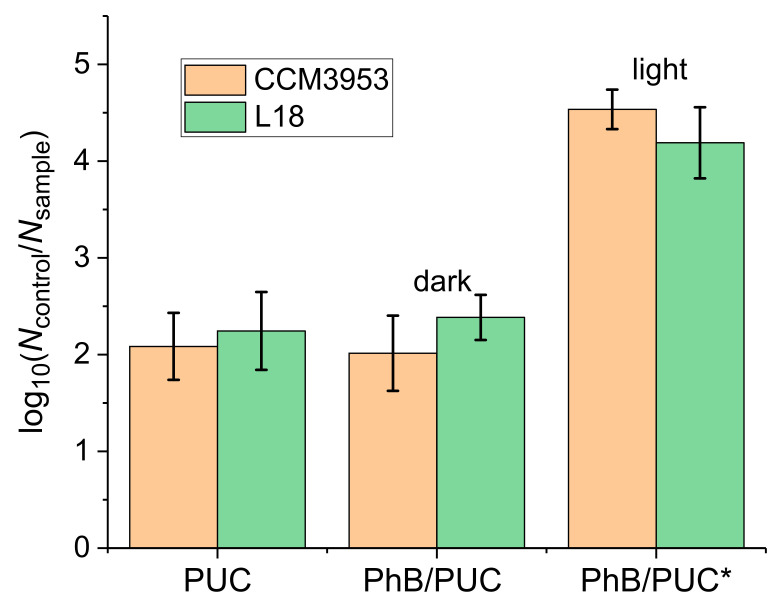
Anti-biofilm effectivity of polyurethane composite (PUC) membranes expressed as the decadic logarithm of cell reduction. The cell number reduction is expressed by the function log(*N*_control_/*N*_sample_), whereby *N*_control_ represents the number of biofilm cells per 1 mL grown in the control group (non-modified PU membrane) (~10^9^); *N*_sample_ represents the number of biofilm cells per 1 mL grown on modified PU membranes (PUC, PhB/PUC); * denotes the sample irradiated with a laser.

The hybrid membranes (PUC, PhB/PUC) were tested for their anti-biofilm properties, as mentioned above. The non-modified PU membrane was used as the control sample. A significant reduction in the survival of biofilm cells (*p*-value ≤ 0.001) with respect to the control sample was observed in all tested composite samples (Figure 8). The presence of PUC and PhB/PUC in the dark reduced the number of biofilm cells by a hundredfold in both CCM 3953 and L 18 strains. Hence, these samples exhibited around 2 × log_10_ reductions, proving a significant antimicrobial efficacy of the membranes even without the application of photodynamic inactivation (PDI), suggesting that the surface properties could contribute to antimicrobial activity. Moreover, the irradiation of the PhB/PUC membrane with a laser led to a further reduction in cell survival (from around 10^9^ cells mL^−1^ to around 10^5^ cells mL^−1^), representing more than a 10,000-fold growth reduction. The reduction due to the irradiation was strongly significant (*p*-value ≤ 0.001). Prolonged irradiation could completely eradicate the cell survival, but the conditions were adjusted to allow the comparison of the efficacy of different membranes. The results in Figure 8 were obtained by the M4 type of membranes. Other types of membranes had significantly lower effectiveness and, therefore, the results are not reported here. The significant reduction of bacterial growth in the presence of PhB has been mentioned by other authors as well. Rasooly et al. [28] received a reduction in survival of Gram-positive bacteria of *Bacillus* sp. and *S. aureus* by 99.99% in the presence of PhB of the concentration of 1.2 mmol L^−1^ and 40 min treatment (reduction from around 10^7^ cells mL^−1^ to around 10^3^ cells mL^−1^). Additionally, they concluded that PhB was active only after irradiation and its antimicrobial activity was dependent on light delivery. This observation is in agreement with our results as PUC and non-irradiated PhB/PUC manifested very similar antimicrobial activity (*p*-value ~ 1), and inhibition was significantly increased with irradiation of PhB/PUC (Figure 8). Another study proved that PhB combined with irradiation had the potential to eradicate the pathogenic caries-causing bacteria present in oral plaque at the hard-to-reach places for normal treatments [29]. After PhB irradiation, singlet oxygen and other reactive oxygen and nitroxide species can be formed that cause damage to cell structures resulting in defects of many vital functions of microorganisms [30]. Another possible mechanism of action of PhB could be due to the reduction in the membrane dipole potential that is dependent on the bilayer composition, resulting in the change of many activities associated with membrane (enzymes or transport) [42]. 

#### 3.4.2. Scanning Electron Microscopy

**Figure 9:**
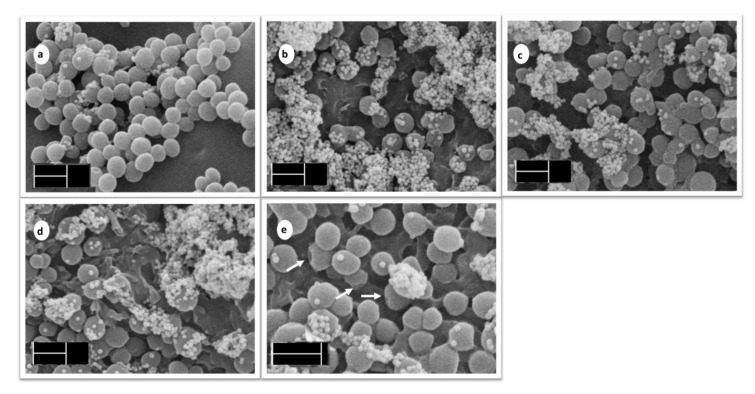
Effectiveness of photodynamic inactivation on MRSA strain *S. aureus* L18. SEM microphotographs are as follows: the 24 h biofilm of *S. aureus* L18 on PU (**a**); PUC (**b**); PhB/PUC (**c**); irradiated PhB/PUC (**d**); detail of biofilm cells on irradiated PhB/PUC (**e**). The magnification was 20,000× (scale 1 µm) for micrographs labeled (**a**–**d**), and 30,000× (scale 1 µm) for micrograph labeled (**e**).

For observation of colonization and architecture of the 24 h biofilms on the tested surfaces, SEM was performed. This method allowed us to evaluate not only biofilm robustness, but also visualize a cell disruption after PDI. Experiments were performed using both the standard CCM 3953 and clinical MRSA isolate L18. As both strains manifested similar results, the micrographs of the standard strain CCM 3953 have been transferred to supplementary data (Figure S7). Figure 9 documents biofilm formed by the MRSA clinical isolate. In the control sample (non-modified PU), the presence of huge clusters of biofilm cells was observed (Figure 9a). In agreement with the already mentioned results from the anti-biofilm assay, the mass of biofilm was markedly reduced on the surfaces of both PUC and PhB/PUC (Figure 9b and 9c, respectively) but also irradiated PhB/PUC (Figure 9d). Moreover, the biofilm cells on irradiated PhB/PUC manifested a higher fraction of the cells with disrupted cell surface (Figure 9e). It is of interest that biofilm cells on all materials produced a huge number of extracellular vesicles (EVs), while a significantly lower number was observed in the control sample (PU; Figure 9a). Production of EVs in *S. aureus* is a phenomenon directly associated with environmental stressors such as antibiotics, iron depletion, or oxidative stress. In such conditions, *S. aureus* yielded more EVs [43]. Observation of EVs on polyurethane composite membranes with modified surfaces is in agreement with previous information as our material combines two important properties which significantly reduce comfort for optimal growth and multiplication of bacteria, namely the anti-adhesive properties of functionalized PU surfaces and oxidative stress arising during irradiation. 

## 4. Conclusions

This work was focused on the development of a new type of polyurethane composite membrane with modified surface. The surface modification was achieved by the presence of organoclay particles, which were functionalized with a photosensitizer, phloxine B. The objective was to achieve anti-biofilm surfaces, mainly in combination with photodynamic inactivation. One of the main benefits was the preparation of a new type of composite material which, unlike conventional polymer composites, contained the particles of a modifier concentrated near the polymer surface. To achieve this, the procedure of polymer synthesis was designed to take place at the interface of the thin film of modified saponite particles and proceed from the liquid phase of the precursors. The polymer chains penetrated the organoclay film before curing the polymer and formed a mixed (composite) phase on the surface of the polymer membrane. This strategy led to the materials exhibiting highly effective surfaces even with the use of a relatively small amount of dispersed active particles. The phase and chemical compositions and properties of the membranes and their surfaces were characterized by X-ray diffraction, IR spectroscopy, and contact angle measurements. By optimizing the composition of the films, it was possible to prepare stable membranes with high content and the controlled release of photosensitizer molecules. Suitable optical properties and photoactivity of the photosensitizer were confirmed by absorption and fluorescence spectroscopy. The reduction of the biofilm cells of *S. aureus* (standard strain and MRSA isolate) was hundredfold for surface-modified PUC membranes. For the membranes containing a photosensitizer and in combination with irradiation, a 4 × log_10_ reduction was achieved. Complete prevention of biofilm growth is possible with prolonged irradiation. The results confirmed the significant anti-biofilm properties and applicability of such composites even to prevent the growth of bacteria resistant to conventional antibiotics. The procedure for the synthesis of polymer composites presented here allows selective modification of surfaces of technical polymers while the polymer matrix remains unchanged. Based on this strategy, it would be possible to prepare modified polymeric materials for practical applications, especially for medical devices.

## Figures and Tables

**Table 1:** The compositions of colloidal dispersions of Sap, ODTMA, and PhB, which were used for the preparation of organoclay films and polyurethane composite membranes tested in preliminary experiments (M1–M4).

Sample	*c*_Sap_	*n*_ODTMA/_ *m*_Sap_	*n*_PhB/_ *m*_Sap_
	g L^−1^	mmol g^−1^	mmol g^−1^
M1	0.1	1.6	0.1
M2	0.1	3.2	0.2
M3	0.1	3.2	0.1
M4	0.1	0.8	0.1

**Table 2:** The amount of absorbed PhB in the films of organoclays M1–M4. The composition of the samples is explained in Table 1. Two replicate tests were done, assigned as (a) or (b). *c*_PhB_^0^, *c*_PhB,supern_, *x*_PhB,%_ represent the initial concentrations of PhB, dye concentration determined in supernatants, and mole fraction of absorbed dye in a membrane expressed in %, respectively. *x*_PhB,%_ was calculated using the formula *x*_PhB,%_ = (*c*_PhB_^0^ − *c*_PhB,supern_) 100%/*c*_PhB_^0^_._

Membrane	*c*_PhB_^0^	*c*_PhB,supern_	*x*_PhB,%_
	(mmol/L)	(mmol/L)	(%)
M1 (a)	0.05	4.0 × 10^−5^	99.9
M1 (b)	0.05	4.5 × 10^−5^	99.9
M2 (a)	0.1	0.0271	72.8
M2 (b)	0.1	0.0262	73.8
M3 (a)	0.05	0.0360	28.1
M3 (b)	0.05	0.0381	23.7
M4 (a)	0.05	6.77 × 10^−4^	98.6
M4 (b)	0.05	6.96 × 10^−4^	98.6

## Data Availability

Data sharing is not applicable.

## Supplementary Materials

The following are available online at https://www.mdpi.com/article/10.3390/ma14247583/s1, Section S1. The effect of the treatment of the medium on PhB properties in the M4 membrane. The methods. Section S2. The release of PhB from the selected polyurethane composite membranes. Section S3. The effect of the treatment of the medium on PhB properties in the M4 membrane. The results. Section S4. X-ray diffraction patterns of selected films. Section S5. Assignment of the bands in infrared spectra. Section S6. Near-infrared spectroscopy. Section S7. Scanning electron microscopy of PU membrane surface. Section S8. Scanning electron microscopy of the biofilm of the standard strain *S. aureus* CCM 3953.
