# Supplementary material for: Surface Characterization and Anti-Biofilm Effectiveness of Hybrid Films of Polyurethane Functionalized with Saponite and Phloxine B
Source: Materials (Basel). 2021 Dec 10;14(24):7583. doi: 10.3390/ma14247583 (PMC8703816; doi:10.3390/ma14247583)
Supplement: Supplementary file 1 [file materials-14-07583-s001.zip › materials-1468120-supplementary.pdf]

Surface Characterization and Anti-Biofilm Effectiveness of Hybrid Films of Polyurethane Functionalized with Saponite and Phloxine B

Nitin Chandra Teja Dadi [1], Juraj Bujdák [2,3,*], Veronika Medvecká [4], Helena Pálková [3], Martin Barlog [3] and Helena Bujdáková [1,*]

[1] Department of Microbiology and Virology, Faculty of Natural Sciences, Comenius University in Bratislava, Ilkovičova 6, 842 15 Bratislava, Slovakia; d1@uniba.sk
[2] Department of Physical and Theoretical Chemistry, Faculty of Natural Sciences, Comenius University in Bratislava, Ilkovičova 6, 842 15 Bratislava, Slovakia
[3] Institute of Inorganic Chemistry, Slovak Academy of Sciences, Dúbravská cesta 9, 845 36 Bratislava, Slovakia; helena.palkova@savba.sk (H.P.); martin.barlog@savba.sk (M.B.)
[4] Department of Experimental Physics, Faculty of Mathematics, Physics and Informatics, Comenius University in Bratislava, 842 48 Bratislava, Slovakia; veronika.medvecka@fmph.uniba.sk
* Correspondence: bujdak3@uniba.sk (J.B.); helena.bujdakova@uniba.sk (H.B.)

Citation: Dadi, N.c.t.; Bujdák, J.; Medvecká, V.; Pálková, H.; Barlog, M.; Bujdáková H. Surface Characterization and Anti-Biofilm Effectiveness of Hybrid Films of Polyurethane Functionalized with Saponite and Phloxine B. *Materials* **2021**, *14*, 7583. https://doi.org/10.3390/ma14247583

Academic Editors: Tomasz Bajda and Jakub Matusik

Received: 1 November 2021
Accepted: 6 December 2021
Published: 10 December 2021

Publisher's Note: MDPI stays neutral with regard to jurisdictional claims in published maps and institutional affiliations.

Copyright: © 2021 by the authors. Submitted for possible open access publication under the terms and conditions of the Creative Commons Attribution (CC BY) license (https://creativecommons.org/licenses/by/4.0/).

Contents

1. The effect of the treatment of the medium on PhB properties in the M4 membrane: The methods.
2. The release of PhB from the selected polyurethane composite membranes.
3. The effect of the treatment of the medium on PhB properties in the M4 membrane: The results.
4. X-ray diffraction patterns of selected films.
5. Assignment of the bands in infrared spectra.
6. Near-infrared spectroscopy.
7. Scanning electron microscopy of PU membrane surface.
8. Scanning electron microscopy of the biofilm of the standard strain *S. aureus* CCM 3953.

S1. The effect of the treatment of the medium on PhB properties in the M4 membrane: The methods

Series of the polymer membranes with PhB/ODTMA/Sap particles (M4 type) were inspected if they exhibit any changes upon contact with the medium MHB used for the biofilm formation. In our previous study, poly(lactic acid) nanocomposites prepared by 3D printing and containing PhB molecules exhibited the release of the dye in various media (Furka et al., 2021). Therefore, the same phenomenon was expected for polyurethane composites. Triplicate polymer membranes were prepared each providing three membrane cuts from the same film, thus having nine specimens to be measured and compared. Another series of nine samples prepared at identical conditions were treated with the medium to check if the properties of the samples changed, namely those related to the photosensitizer.

Absorption and fluorescence spectroscopies were applied to characterize the properties of PhB. Vibrational spectroscopy methods (IR and NIR) were measured to characterize the properties of the surfactant and polymer. The spectral data matrices were decomposed using principal component analysis (PCA) and multivariate curve resolution (MCR) (Unscrambler, Camo). More about the use of the methods is provided in specialized literature. The MCR spectra were normalized to a unity vector and concentrations obtained from the MCR analysis were used to compare the differences in the intensity of emitted light. The sum of the concentrations of both the components was used in the student test.

References:

[24] Furka, S.; Furka, D.; Dadi, N. C. T. C. T.; Palacka, P.; Hromníková, D.; Dueñas Santana, J. A.; Pineda, J. D.; Casas, S. D.; Bujdák, J. Novel Antimicrobial Materials Designed for the 3d Printing of Medical Devices Used During the Covid-19 Crisis. *Rapid Prototyping Journal* **2021,** *27*, 890-904.

S2. The release of PhB from the selected polyurethane composite membranes.

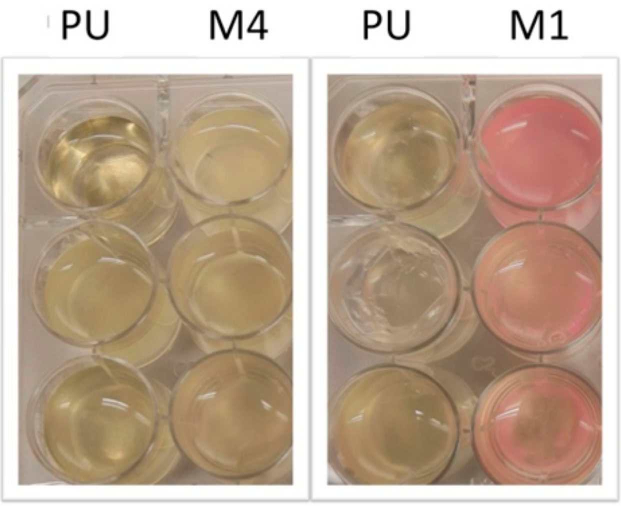

Figure S1. The microtitre plates with Mueller-Hinton broth released phloxine B from the treated membranes during the formation of *S. aureus* biofilm (PU—polyurethane membrane, PhB/PUC: M4 and M1).

Three replicas of the membranes M1 and M4 were treated in the same way and under the same conditions as used for biofilm growth but without the presence of microorganisms. Non-modified polyurethane was used as the control. After 24 h, the films were removed from the wells of microtiter plates and the coloring was documented. Only a little coloring by PhB was observed for the dye release from the M4 membrane. On the other hand, a significant release of the dye was observed for the M1 membrane.

S3. The effect of the treatment of the medium on PhB properties in the M4 membrane. The results.

Fluorescence spectroscopy was used as the most sensitive method to detect the amount and activity of fluorophores. A relatively broad range of the maxima for PhB absorption was observed (590 nm ± 10 nm) which can be assigned to the heterogeneity of the samples contributing to the factors which sensitively affect the energy of emitted light (energy migration, transfer, light scattering, and reabsorption, etc). MCR method could identify two main components emitting at 582 and 595 nm.

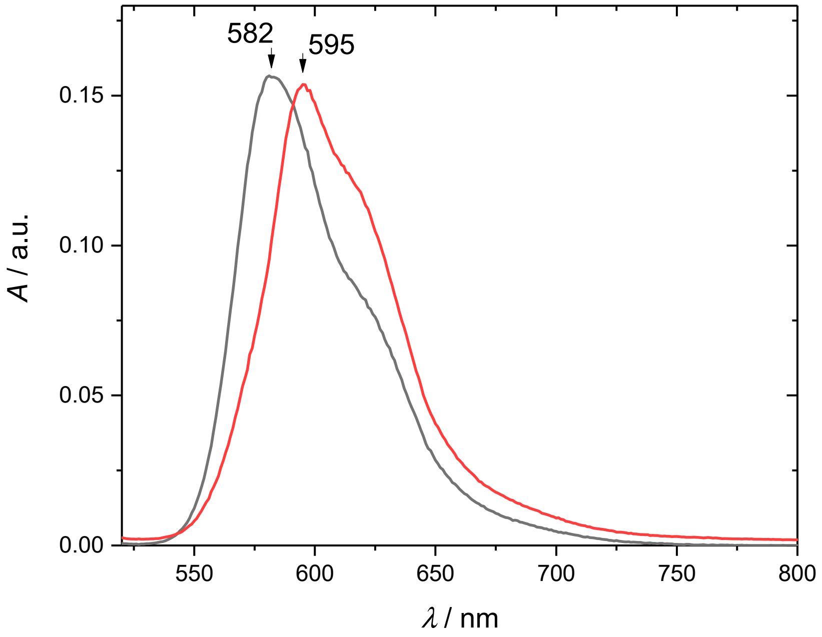

Figure S2a. Two main components in PhB emission spectra identified by the MCR method.

PCA method was able to identify two PhB spectral components from the reconstruction of the series of fluorescence spectra (Figure S3b). PCA method supports the results of MCR. The loadings provided information on the spectral profiles of the dye forms in the samples. The main component assigned as PC-1 expressed an average PhB spectrum with an emission maximum at 592 nm. The main component PC-1 representing an average spectrum exhibited significantly higher emission for the group of untreated samples. The identity of at least two spectral forms is indicated by the shape of PC-2 loading with positive and negative bands at 575 and 600 nm, respectively. It means, two dye forms emitting at higher and lower wavelengths could be identified. (More accurate analysis of the spectra using the MCR method proved the presence of the species emitting at 582 and 595 nm, respectively (Figure S3a)). The scores identified significant differences between the groups of treated and untreated samples. (Figure S3b, lower). The main component PC-1 exhibited much higher emission for the group of untreated samples, which can be interpreted by a partial release of the dye from the samples upon the treatment. The component PC-2 did not depend significantly on the treatment of the samples. It means the treatment did not bring the qualitative spectral changes but only the reduction of emission intensity.

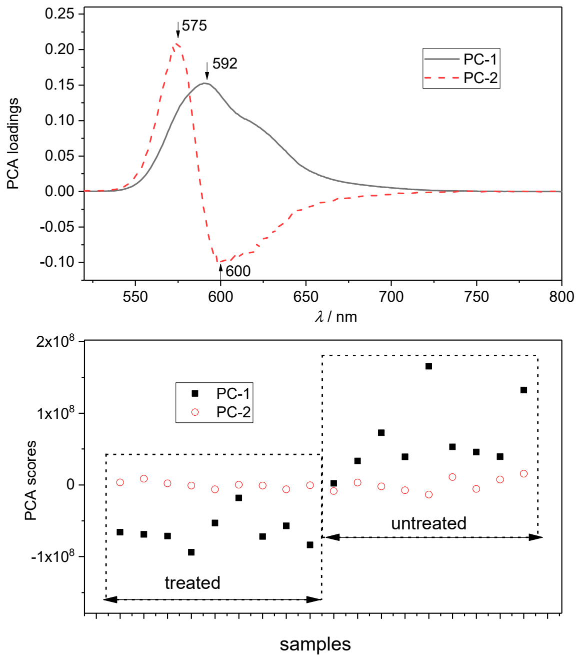

Figure S2b. The results of principal component analysis of fluorescence spectral data. The loadings and scores represent the spectral profile and concentration of the species, respectively.

Chemometric analysis of the data obtained from vibrational spectroscopy did not prove the release of the surfactant cations from the membranes of polyurethane composites upon contact with the medium.

S4. X-ray diffraction patterns of selected films.

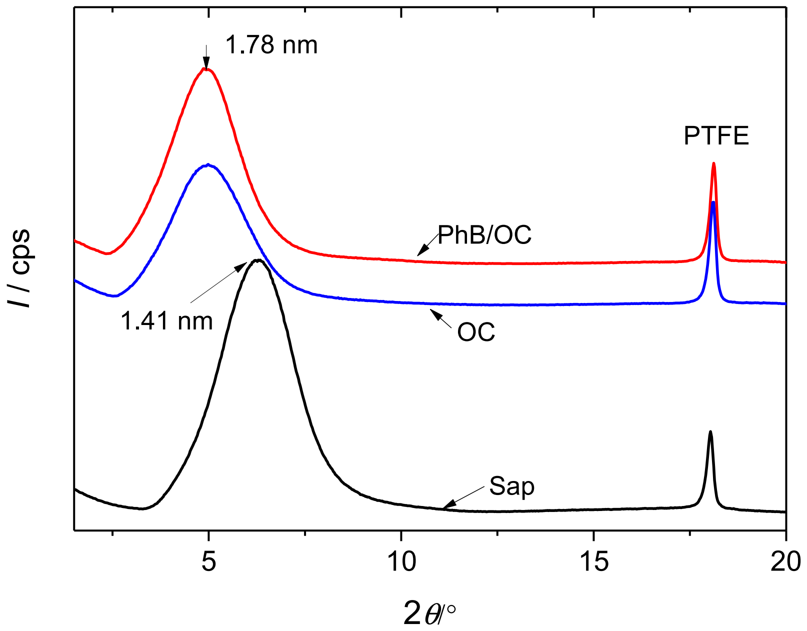

Figure S3. X-ray diffraction patterns of saponite (Sap) and organoclay film (OC), and organoclay functionalized with phloxine B (PhB/OC). The labels denote the values of 001 spacings. The signal of polytetrafluoroethylene (PTFE) membrane was also observed at 17.8 °.

S5. Assignment of the bands in infrared spectra.

Table S1. Assignment of the bands in infrared spectra.

Band wavenumber (cm^{-1})				Assignement
PU	PTFE	OC-PU	OC	
		3682	3682	νOH –structural OH groups of saponite
3527				νOH – water molecules
3384		3380		νNH – H-bonded
2955				νCH – asymmetric O–CH_2 groups
2934		2935	2935	νCH – asymmetric from CH_2 groups
		2926	2926	νCH – asymmetric from CH_2 groups
2865		2854, 2870	2854, 2870	νCH – symmetric from CH_2 groups
1726		1728, 1723		νC=O – no H-bonded
1688		1687		νC=O – H-bonded
1538		1557, 1537		δNH – amide II, trans form
1457		1454, 1464	1454, 1464	δCH – from CH_2 groups
1240		1249, 1240		stretching, bending C-C
	1203			νCF – from CF_2 groups
	1150			νCF – from CF_2 groups
1147		1147		νCO – from C-O-C-N
1073				νCO – from C-O-C-N
1024				stretching, bending C-C
		985	985	νSiO asymmetric
846				stretching, bending C-C
763		766		stretching, bending C-C
		693	693	SiO translational from saponite
		658		δOH – structural OH groups of saponite
	640			δC-C-F, PTFE
	554			δC-C-F, PTFE
	505			δC-C-F, PTFE
		432	432	δSiO – saponite

ν—stretching vibration, δ—bending vibration.

S6. Near-infrared spectroscopy.

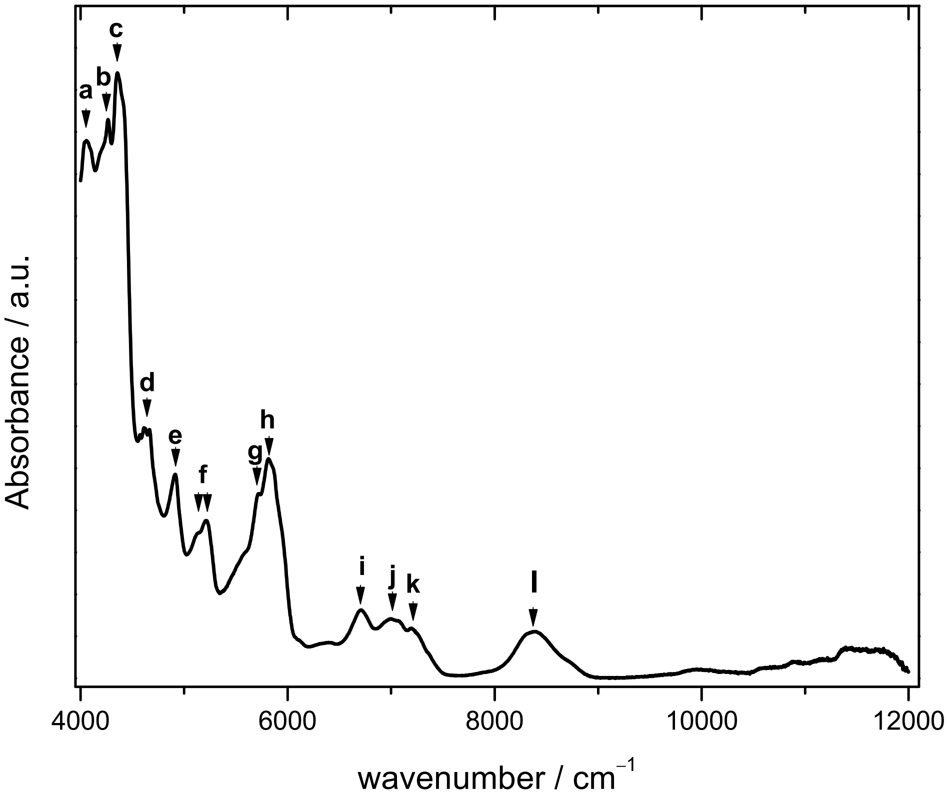

Figure S5. Near-infrared spectrum of a polyurethane membrane and assignment of the spectral bands. The assignments of the bands: a. $(\nu_a+\delta_r)$(C-H), 4055 cm^{-1}; b. $(\nu_s+\delta)$(C-H), 4265 + ~4193 cm^{-1}; c. $(\nu+\delta)$(C-H), 4360 cm^{-1}; d. 2ν(C=O)+δ(C-N), 4630 cm^{-1}; ν(NH)+δ(C-N), 4665 + 4620 cm^{-1}, e. f. 3ν(C=O), 4910 cm^{-1}; g. $(\nu_a+\nu_a)$(C-H), 5715+~5575 cm^{-1}; h. $(\nu_a+\nu_s)$(C-H), 5820+~5865 cm^{-1}; j. $(2\nu+\delta)$(C-H), (2ν)(N-H),7000 +~7075 cm^{-1}; k. $(2\nu+\delta)$(C-H), 7205 cm^{-1}; l. (3ν)(C-H), 8380 +~8730 cm^{-1}. The spectra of composites exhibited almost an identical spectral profile (not shown).

S7. Scanning electron microscopy of PU membrane surface

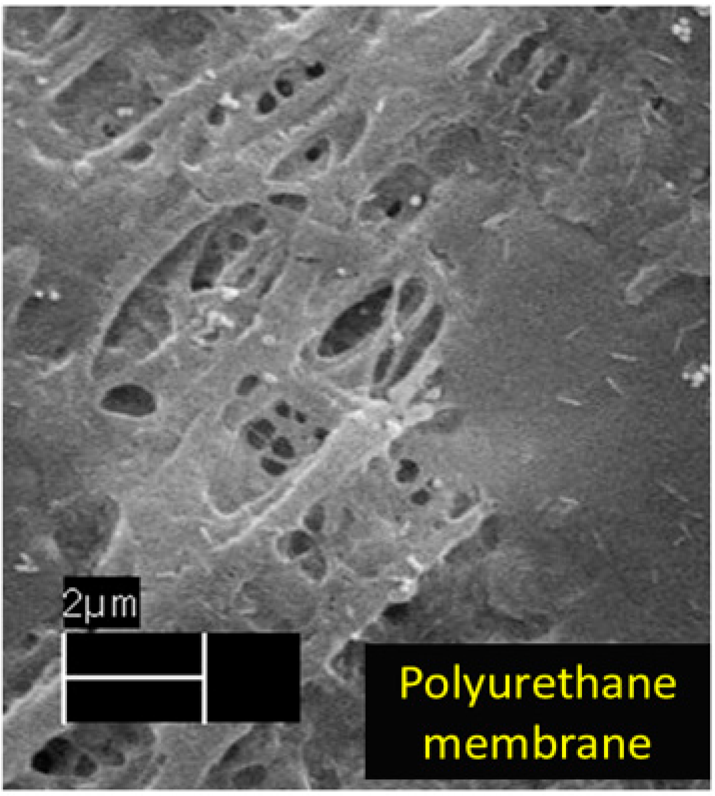

Figure S6. Surface topography with microcavities occurring on some parts of polyurethane membrane surface by SEM.

S8. Scanning electron microscopy of the biofilm of the standard strain *S. aureus* CCM 3953.

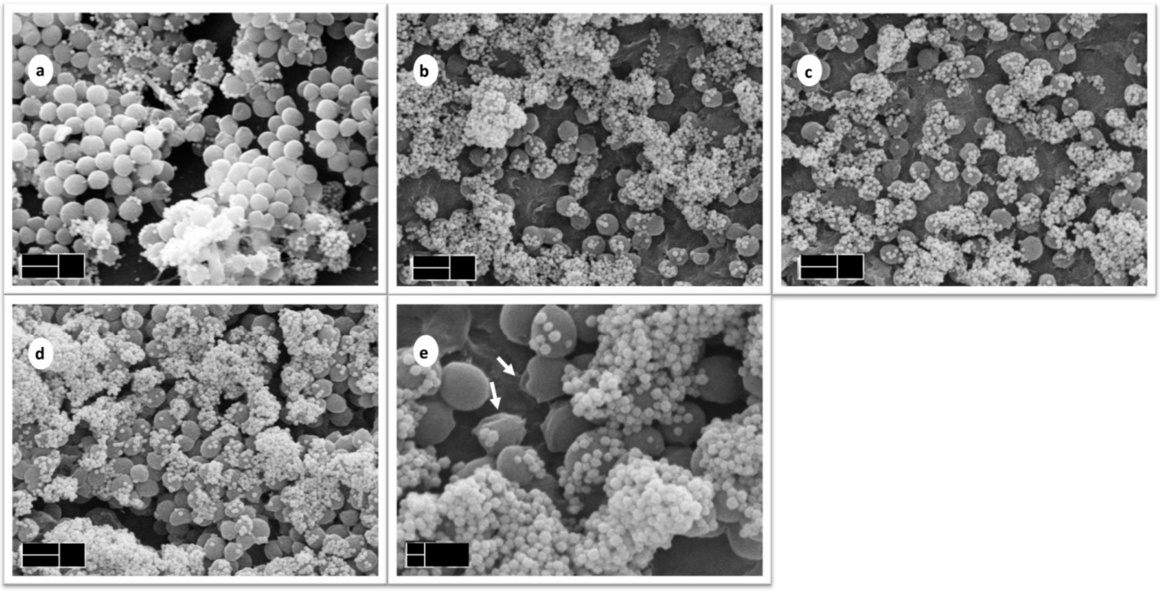

Figure S7. Effectiveness of photodynamic inactivation on the standard strain *S. aureus* CCM 3953. SEM microphotographs are as follows: the 24-h biofilm of *S. aureus* CCM 3953 on PU (a); PUC (b); PhB/PUC (c); irradiated PhB/PUC (d); detail of biofilm cells on irradiated PhB/PUC (e). The magnification was 20 000 x (scale 1 μm) for micrographs labeled a; b; c; d, and 50 000 x (scale 0.2 μm) for micrograph labeled e. The white arrows point to the sites, where cell damage was observed.
